# Author Correction: Interplay of protein corona and immune cells controls blood residency of liposomes

**DOI:** 10.1038/s41467-020-15500-9

**Published:** 2020-03-31

**Authors:** Francesca Giulimondi, Luca Digiacomo, Daniela Pozzi, Sara Palchetti, Elisabetta Vulpis, Anna Laura Capriotti, Riccardo Zenezini Chiozzi, Aldo Laganà, Heinz Amenitsch, Laura Masuelli, Giovanna Peruzzi, Morteza Mahmoudi, Isabella Screpanti, Alessandra Zingoni, Giulio Caracciolo

**Affiliations:** 1 Department of Molecular Medicine, Sapienza University of Rome, Viale Regina Elena 291, 00161 Rome, Italy; 2 Istituto Italiano di Tecnologia, Center for Life Nano Science@Sapienza, Viale Regina Elena 291, 00161 Rome, Italy; 3 Department of Chemistry, Sapienza University of Rome, P.le Aldo Moro 5, 00185 Rome, Italy; 4 Institute of inorganic Chemistry, Graz University of Technology, Stremayerg 6/IV, 8010 Graz, Austria; 5 Department of Experimental Medicine, Sapienza University of Rome, Viale Regina Elena 324, 00161 Rome, Italy; 6 Precision Health Program, Michigan State University, East Lansing, MI 48823 USA

**Keywords:** Nanobiotechnology, Nanomedicine, Proteomics

Correction to: *Nature Communications* 10.1038/s41467-019-11642-7, published online 15 August 2019.

The original version of this Article omitted from the author list the 11th author ‘Giovanna Peruzzi’, who is from the ‘Istituto Italiano di Tecnologia, Center for Life Nano Science@Sapienza, Viale Regina Elena 291, 00161, Rome, Italy’. Consequently, the new author list and affiliations is as follows;

“Francesca Giulimondi^1,2,7^, Luca Digiacomo^1,7^, Daniela Pozzi^1^, Sara Palchetti^1^, Elisabetta Vulpis^1^, Anna Laura Capriotti^3^, Riccardo Zenezini Chiozzi^3^, Aldo Laganà^3^, Heinz Amenitsch^4^, Laura Masuelli^5^, Giovanna Peruzzi^2^, Morteza Mahmoudi^6^, Isabella Screpanti^1^, Alessandra Zingoni^1^ & Giulio Caracciolo^1^

^1^Department of Molecular Medicine, Sapienza University of Rome, Viale Regina Elena 291, 00161 Rome, Italy. ^2^Istituto Italiano di Tecnologia, Center for Life Nano Science@Sapienza, Viale Regina Elena 291, 00161, Rome, Italy. ^3^Department of Chemistry, Sapienza University of Rome, P.le Aldo Moro 5, 00185 Rome, Italy. ^4^Institute of inorganic Chemistry, Graz University of Technology, Stremayerg 6/IV, 8010 Graz, Austria. ^5^Department of Experimental Medicine, Sapienza University of Rome, Viale Regina Elena 324, 00161 Rome, Italy. ^6^Precision Health Program, Michigan State University, East Lansing, MI 48823, USA. ^7^These authors contributed equally: Francesca Giulimondi, Luca Digiacomo. Correspondence and requests for materials should be addressed to M.M. (email: mahmou22@msu.edu) or to G.C. (email: giulio.caracciolo@uniroma1.it)”

This has been corrected in both the PDF and HTML versions of the Article.

