# Supplementary material for: Interplay of protein corona and immune cells controls blood residency of liposomes
Source: Nat Commun. 2019 Aug 15;10:3686. doi: 10.1038/s41467-019-11642-7 (PMC6695391; doi:10.1038/s41467-019-11642-7)
Supplement: Supplementary file 1 — Supplementary Information [file 41467_2019_11642_MOESM1_ESM.pdf]

Interplay of protein corona and immune cells controls blood residency of liposomes

Giulimondi et al.

A

SAMPLE	MW	$[M+H]^+$	$[M-H]^-$	S-LENS	PRUDUCT	COLLISION ENERGY (V)
DOTAP	662.6	663.6	-	260.3	95.2	45
					134.9	33
					603.5	23
DOPC	786.1	-	785.1	255.4	78.9	50
					168.0	34
					281.2	26
DOPG	797.0	-	796.0	250.2	78.9	50
					171.0	35
					281.2	27

B

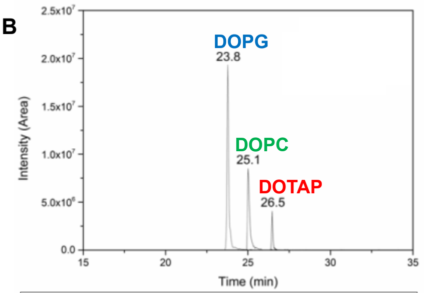

C

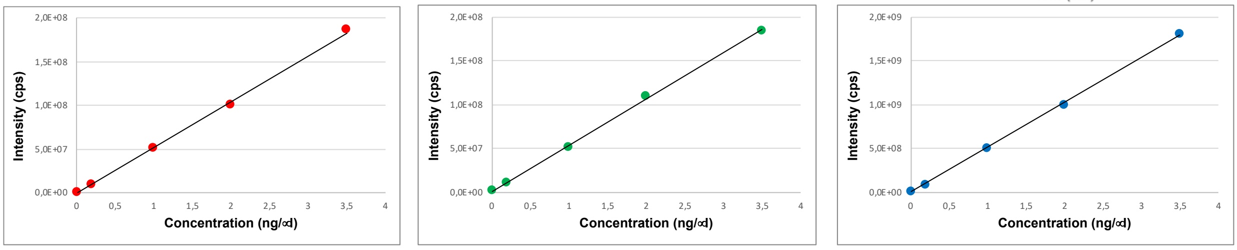

D

	DOTAP (ng/µL)		DOPC (ng/µL)		DOPG (ng/µL)	
	HP=5%	HP=50%	HP=5%	HP=50%	HP=5%	HP=50%
surnatant	35,33 ± 5,64	26,46 ± 4,78	42,76 ± 3,54	28,90 ± 3,08	40,44 ± 3,26	32,12 ± 5,21
washing 1	0,56 ± 0,01	0,22 ± 0,09	0,99 ± 0,08	0,47 ± 0,13	0,84 ± 0,19	0,25 ± 0,12
washing 2	0,32 ± 0,02	0,17 ± 0,11	0,52 ± 0,10	0,25 ± 0,07	0,43 ± 0,13	0,21 ± 0,11
washing 3	0,24 ± 0,04	0,16 ± 0,09	0,20 ± 0,09	0,15 ± 0,11	0,28 ± 0,09	0,14 ± 0,09

E

	DOTAP (%)		DOPC (%)		DOPG (%)	
	HP=5%	HP=50%	HP=5%	HP=50%	HP=5%	HP=50%
pellet	96,4 ± 0,6	97,3 ± 0,5	95,6 ± 0,4	97,0 ± 0,3	95,8± 0,3	96,7 ± 0,5

Figure 1. Multiple reaction monitoring (MRM) transitions (A) and chromatograms (B) of DOTAP, DOPC and DOPG. (C) Calibration curves for DOTAP (red points), DOPC (green points) and DOPG (blue points) in the concentration range between 0,01 ng/µl and 3,5 ng/µl. Solid lines represent the best linear fits to the data. R^2 values were: 0.9985 (DOTAP), 0,9995 (DOPC) and 0,9991 (DOPG). (D) Liposome-protein complexes were isolated by centrifugation for 15 min at 14,000 rpm. Then, pellets were washed three times with PBS to remove unbound and loosely bound proteins (the 'soft corona') obtaining the so-called 'hard corona'. Lipid concentration was calculated in the supernatant (i.e. after centrifugation) as well as after each of the three washing steps. When lipid concentration was out of the linear range (i.e. higher than 3.5 ng/µL), samples were pre-diluted. Dilution factors were considered to estimate lipid concentration. (E) Lipid percentage in lipid-protein complexes with respect to bare liposomes (i.e. before exposure to HP). Results are provided as average ± standard deviation of three replicates.

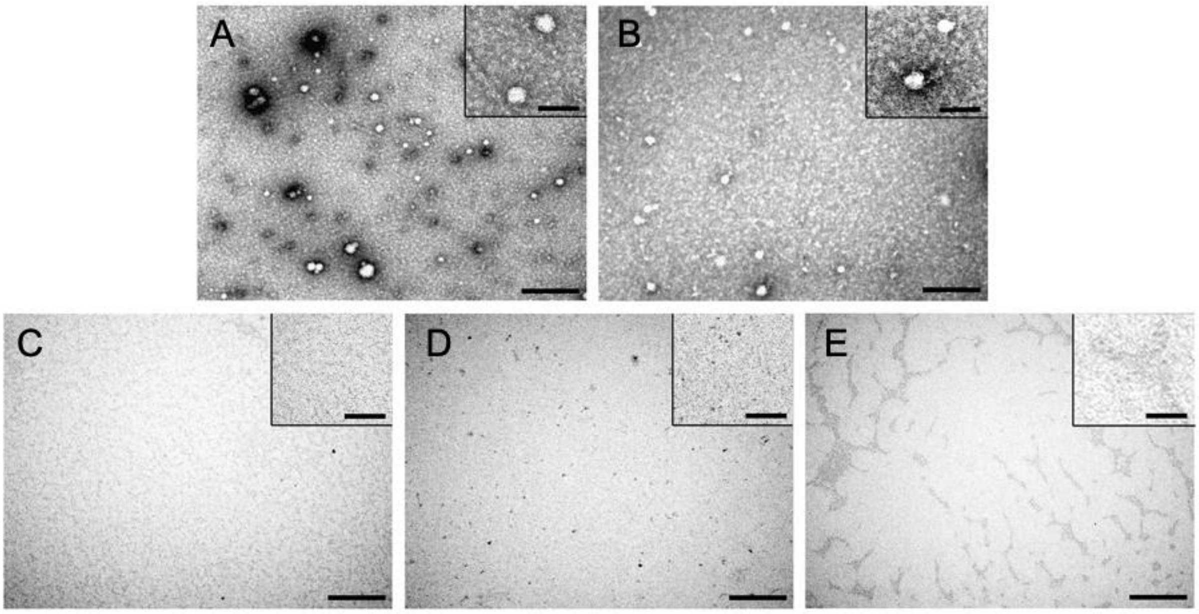

Figure 2. (A) Representative transmission electron microscopy (TEM) image of DOTAP-protein complexes after 1h incubation with 50% human plasma before centrifugation. Then, liposome-protein complexes were isolated by centrifugation for 15 min at 14,000 rpm. In most cases, TEM images of the supernatant did not contain any trace of vesicles (not reported). When vesicle-like structures were seen (B), they were prevalently bare liposomes). This suggests that the minor fraction of lipids found in the supernatant (as determined by UHPLC/ESI-MS/MS), was due to uncoated liposomes (i.e. in the absence of biomolecular corona). Then, pellets were washed three times with PBS. TEM images were acquired on the washing buffer after each of the three washing steps (C-E). As evident, no plain evidence of liposome-protein complexes was found. We only detected a weak signal likely due to free proteins. Figure legend: scale bars correspond to 200nm. Scale bars in the insets correspond to 100 nm.

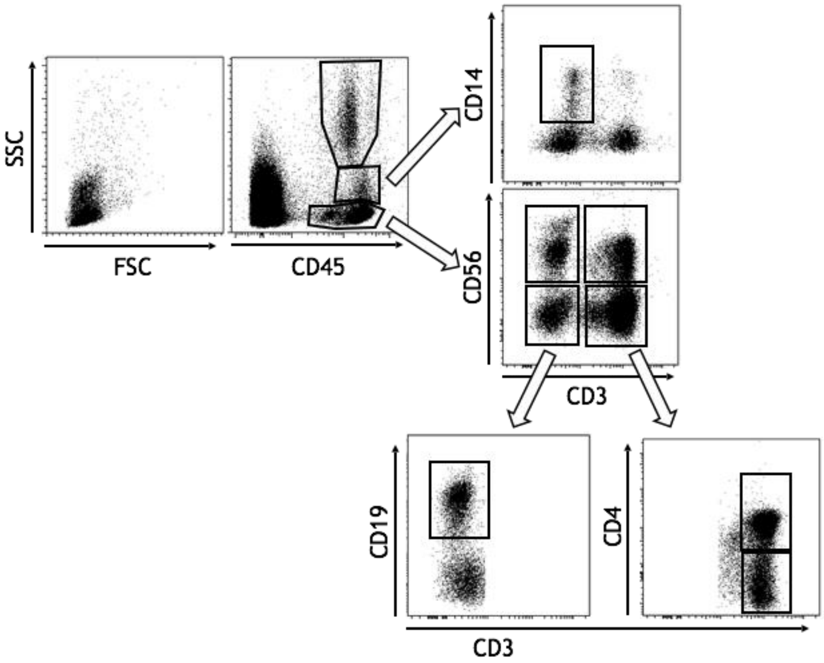

Figure 3: Gating strategy of distinct leukocyte subpopulations. Representative dot plots of leukocyte subpopulations identified by gating on CD45⁺ cells and dissected based on the expression of CD14, CD3, CD56, CD19 and CD14 surface markers as indicated. Granulocytes were identified based on the high SSC parameter.
Gating panels correspond to flow cytometry data reported in panels A, C and E of Figure 5.

Table 1: **Liposome characterization.** Hydrodynamic diameter (D_H), polydispersity index (PdI) and zeta potential (Z_p) of bare DOTAP, DOPC and DOPG liposomes. Each value is the average of triplicate experiments ± standard deviation.

	**D_H (nm)**	**PdI**	**Z_p (mV)**
**DOTAP**	149.1±0.8	0.17±0.08	51.4±0.7
**DOPC**	108.2±0.5	0.22±0.06	-0.5±0.1
**DOPG**	124.8±0.6	0.06±0.02	-49.5±0.8

Table 2. Protein bound to liposomes' coronas. Nanograms of protein per milligram of lipid**.** Each value is the average of triplicate experiments ± standard deviation.

HP(%)	DOTAP	DOPC	DOPG
**1**	3.0±0.1	0.22±0.01	0.28±0.01
**2.5**	6.2±1.1	0.24±0.02	0.50±0.02
**5**	10.7±0.2	0.15±0.05	0.80±0.02
**10**	15.6±3.3	0.52±0.11	1.03±0.04
**20**	12.2±0.3	1.36±0.07	1.42±0.05
**50**	15.8±5.2	1.33±0.01	2.53±0.02

Table 3. Proteins identified in the respective liposomes' coronas by nano-LC MS/MS.

PROTEIN					Relative Protein Abundance (%)					
					DOTAP		DOPC		DOPG	
	Accession number	Mol. weight [kDa]	Isoelectric point	Category	HP=5%	HP=50%	HP=5%	HP=50%	HP=5%	HP=50%
**1**	A1AG1	23,51	4,66	Acute Phase	0,11	0,04	0,28	0,31	0,31	0,37
**2**	A1AG2	23,60	4,76	Acute Phase	0,12	0,01	0,18	0,21	0,15	0,18
**3**	AACT	47,65	5,19	Acute Phase	0,10	0,01	0,06	0,05	0,08	0,05
**4**	A1AT	46,74	5,31	Acute Phase	3,59	0,87	0,91	0,77	0,94	1,02
**5**	A1BG	54,25	5,72	Acute Phase	0,10	0,01	0,03	0,03	0,04	0,02
**6**	A2MG	163,29	6,43	Acute Phase	0,21	0,10	0,22	0,11	0,14	0,11
**7**	HPT	45,21	6,56	Acute Phase	1,94	0,38	1,96	1,36	1,45	1,02
**8**	HPTR	39,03	7,10	Acute Phase	0,19	0,12	0,08	0,15	0,09	0,07
**9**	LBP	53,38	6,68	Acute Phase	0,00	0,06	0,10	0,10	0,17	0,33
**10**	SAA1	13,53	8,14	Acute Phase	0,16	0,02	0,00	0,01	0,02	0,05
**11**	SAA4	14,75	9,39	Acute Phase	0,09	0,01	0,07	0,21	0,19	0,23
**12**	A2AP	54,57	6,24	Coagulation	0,07	0,06	0,01	0,04	0,02	0,02
**13**	ANT3	52,60	6,68	Coagulation	0,05	0,00	0,03	0,02	0,04	0,02
**14**	FA9	51,78	5,16	Coagulation	0,01	0,59	0,00	0,00	0,00	0,00
**15**	FA5	251,70	5,94	Coagulation	0,00	0,08	0,09	0,00	0,06	0,15
**16**	FA7	51,59	7,23	Coagulation	0,00	0,03	0,00	0,00	0,00	0,00
**17**	FA10	54,73	5,74	Coagulation	0,00	0,28	0,00	0,00	0,00	0,01
**18**	FA11	70,11	8,14	Coagulation	0,00	0,00	0,00	0,00	0,04	0,03
**19**	FA12	67,79	7,76	Coagulation	0,00	0,00	0,00	0,00	0,07	0,00
**20**	F13A	83,27	5,94	Coagulation	0,00	0,09	0,00	0,00	0,00	0,00
**21**	FIBA	94,97	5,87	Coagulation	4,58	6,80	1,53	1,51	2,86	1,61
**22**	FIBB	55,93	8,39	Coagulation	7,72	11,05	2,77	3,19	5,29	3,54
**23**	FIBG	51,51	5,32	Coagulation	5,22	10,16	2,15	2,60	3,57	2,53
**24**	FINC	262,62	5,45	Coagulation	0,46	0,16	0,03	0,04	0,05	0,05
**25**	KNG1	71,96	6,81	Coagulation	0,32	0,92	0,13	0,11	0,52	0,57
**26**	THRB	70,04	5,70	Coagulation	1,36	5,92	0,28	0,19	0,03	0,28
**27**	PROC	52,07	6,22	Coagulation	0,00	0,28	0,00	0,00	0,00	0,00
**28**	PROS	75,12	5,37	Coagulation	0,05	0,66	0,17	0,01	0,03	0,02
**29**	PROZ	44,74	5,87	Coagulation	0,00	0,15	0,00	0,00	0,00	0,00
**30**	C1QA	26,02	9,46	Complement	0,00	0,02	0,61	0,02	0,80	0,27
**31**	C1QB	26,72	8,87	Complement	0,05	0,03	1,03	0,03	1,39	0,74
**32**	C1QC	25,77	8,58	Complement	0,18	0,16	0,83	0,03	1,42	0,44
**33**	C1QT3	26,99	6,50	Complement	0,00	0,00	0,00	0,00	0,00	0,00
**34**	C1R	80,12	6,15	Complement	0,14	0,20	0,11	0,00	0,35	0,07
**35**	C1RL	53,50	7,21	Complement	0,00	0,00	0,00	0,00	0,00	0,00
**36**	C1S	76,68	4,58	Complement	0,08	0,15	0,09	0,01	0,24	0,06
**37**	CO2	83,27	7,44	Complement	0,01	0,00	0,00	0,00	0,00	0,00
**38**	CO3	187,15	6,34	Complement	1,37	1,08	1,07	2,36	1,30	1,74
**39**	CO4A	192,78	7,07	Complement	0,00	0,08	0,04	0,03	0,02	0,03
**40**	CO4B	192,75	7,28	Complement	1,14	2,59	2,03	0,59	0,78	0,61
**41**	CO5	188,30	6,49	Complement	0,05	0,46	0,01	0,06	0,05	0,03
**42**	CO6	104,79	6,74	Complement	0,02	0,00	0,00	0,00	0,00	0,00
**43**	CO7	93,52	6,44	Complement	0,00	0,00	0,00	0,00	0,00	0,00
**44**	CO8A	65,16	6,42	Complement	0,01	0,00	0,00	0,00	0,02	0,00
**45**	CO8B	67,05	8,17	Complement	0,00	0,00	0,00	0,00	0,09	0,01
**46**	CO8G	22,28	8,48	Complement	0,00	0,00	0,00	0,00	0,07	0,01
**47**	CO9	63,17	5,27	Complement	0,16	0,14	0,01	0,03	0,02	0,02
**48**	CFAB	85,53	7,07	Complement	0,20	0,08	0,03	0,01	0,11	0,02
**49**	CFAD	27,03	7,78	Complement	0,00	0,00	0,00	0,00	0,10	0,04
**50**	CFAH	139,09	6,59	Complement	0,21	0,06	0,09	0,05	0,47	0,29
**51**	FHR1	37,65	7,42	Complement	0,00	0,00	0,13	0,00	0,64	0,85
**52**	FHR2	30,65	6,34	Complement	0,00	0,00	0,00	0,00	0,03	0,02
**53**	FHR4	65,35	4,57	Complement	0,00	0,00	0,00	0,01	0,00	0,00
**54**	FHR5	64,42	7,07	Complement	0,00	0,00	0,02	0,02	0,07	0,17
**55**	CFAI	65,75	7,53	Complement	0,00	0,03	0,00	0,00	0,01	0,00
**56**	IGA2	48,93	6,23	Immunoglobulins	0,23	0,08	0,12	0,18	0,09	0,07
**57**	IGHA1	37,65	6,49	Immunoglobulins	1,99	0,77	1,18	1,43	1,09	0,94
**58**	IGD	56,22	8,02	Immunoglobulins	0,01	0,00	0,00	0,00	0,01	0,00
**59**	IGG1	49,33	8,30	Immunoglobulins	1,32	1,65	4,71	1,98	2,99	2,17
**60**	IGHG2	35,90	7,66	Immunoglobulins	0,14	0,26	1,01	0,70	0,77	0,53
**61**	IGHG3	41,29	7,95	Immunoglobulins	0,13	0,15	1,49	0,44	1,24	0,57
**62**	IGHG4	35,94	7,41	Immunoglobulins	0,04	0,01	0,08	0,04	0,16	0,07
**63**	IGHM	49,44	6,77	Immunoglobulins	1,73	0,65	0,87	3,70	0,98	0,94
**64**	HV108	12,99	8,92	Immunoglobulins	0,00	0,00	0,02	0,00	0,00	0,00
**65**	HV315	12,93	8,84	Immunoglobulins	0,00	0,00	0,07	0,03	0,02	0,01
**66**	HV335	12,95	9,17	Immunoglobulins	0,00	0,00	0,04	0,05	0,04	0,00
**67**	HV349	13,06	8,81	Immunoglobulins	0,00	0,00	0,02	0,01	0,01	0,01
**68**	HV307	12,94	8,32	Immunoglobulins	0,01	0,01	0,03	0,20	0,04	0,05
**69**	HV374	12,84	8,57	Immunoglobulins	0,06	0,03	0,14	0,10	0,08	0,06
**70**	HV428	13,12	9,59	Immunoglobulins	0,00	0,00	0,01	0,00	0,00	0,00
**71**	HV404	12,85	10,05	Immunoglobulins	0,00	0,00	0,01	0,04	0,02	0,00
**72**	IGJ	18,10	4,86	Immunoglobulins	0,16	0,03	0,11	0,39	0,09	0,13
**73**	IGKC	11,77	5,68	Immunoglobulins	0,73	0,32	1,17	0,59	0,92	0,37
**74**	IGK	23,38	6,90	Immunoglobulins	3,43	1,22	2,40	2,77	2,03	1,80
**75**	KV133	12,85	5,35	Immunoglobulins	0,02	0,00	0,00	0,00	0,00	0,00
**76**	KV105	12,78	8,46	Immunoglobulins	0,03	0,00	0,00	0,00	0,00	0,00
**77**	KV224	13,08	8,73	Immunoglobulins	0,01	0,00	0,00	0,03	0,01	0,00
**78**	KV228	12,96	6,49	Immunoglobulins	0,16	0,00	0,04	0,00	0,00	0,00
**79**	KV315	12,50	7,43	Immunoglobulins	0,18	0,00	0,00	0,04	0,07	0,00
**80**	KV320	12,56	9,49	Immunoglobulins	0,02	0,00	0,29	0,28	0,08	0,10
**81**	KVD11	12,63	4,76	Immunoglobulins	0,04	0,00	0,09	0,02	0,07	0,03
**82**	KVD15	12,53	4,85	Immunoglobulins	0,01	0,00	0,01	0,00	0,01	0,00
**83**	KV401	13,38	4,85	Immunoglobulins	0,14	0,00	0,07	0,06	0,05	0,04
**84**	IGLC3	11,27	7,34	Immunoglobulins	0,92	0,12	1,41	0,60	0,74	0,36
**85**	IGLC7	11,25	8,48	Immunoglobulins	0,00	0,00	0,01	0,00	0,00	0,00
**86**	LV140	12,30	9,62	Immunoglobulins	0,02	0,00	0,00	0,00	0,00	0,00
**87**	LV147	12,28	6,99	Immunoglobulins	0,09	0,00	0,04	0,00	0,03	0,00
**88**	LV151	12,25	8,21	Immunoglobulins	0,00	0,00	0,03	0,01	0,02	0,01
**89**	LV310	12,44	4,65	Immunoglobulins	0,00	0,00	0,00	0,00	0,01	0,01
**90**	LV319	12,04	6,50	Immunoglobulins	0,00	0,00	0,01	0,01	0,02	0,01
**91**	LV321	12,45	6,11	Immunoglobulins	0,48	0,03	0,04	0,00	0,02	0,02
**92**	LV469	12,77	6,49	Immunoglobulins	0,00	0,00	0,00	0,00	0,00	0,00
**93**	LV539	13,39	8,03	Immunoglobulins	0,00	0,00	0,00	0,00	0,00	0,00
**94**	LV746	12,47	7,20	Immunoglobulins	0,00	0,00	0,01	0,02	0,03	0,00
**95**	LV949	13,02	7,41	Immunoglobulins	0,00	0,00	0,00	0,00	0,00	0,00
**96**	IGL1	22,83	8,55	Immunoglobulins	2,07	0,72	2,36	2,01	1,61	1,33
**97**	APOA1	30,78	5,50	Lipoproteins	10,38	2,03	3,53	7,01	5,91	8,16
**98**	APOA2	11,18	6,61	Lipoproteins	5,80	3,00	2,70	3,08	3,02	4,43
**99**	APOA4	45,40	5,04	Lipoproteins	1,67	0,95	0,34	0,38	0,41	0,30
**100**	APOB	515,60	7,04	Lipoproteins	0,33	0,84	0,69	2,32	0,87	1,54

**101**	APOC1	9,33	9,18	Lipoproteins	2,91	0,05	2,62	4,20	0,11	2,29
**102**	APOC2	11,28	4,37	Lipoproteins	2,63	3,18	1,04	5,94	2,84	5,14
**103**	APOC3	10,85	5,04	Lipoproteins	3,79	3,65	2,11	5,50	6,61	9,52
**104**	APOC4	14,55	9,14	Lipoproteins	0,17	0,00	0,00	0,15	0,01	0,21
**105**	APOD	21,28	4,80	Lipoproteins	0,15	0,19	0,37	0,36	0,54	0,29
**106**	APOE	36,15	5,42	Lipoproteins	0,66	1,09	0,89	2,47	1,34	2,81
**107**	APOF	35,40	5,36	Lipoproteins	0,01	0,26	0,03	0,04	0,02	0,03
**108**	APOL1	43,97	5,55	Lipoproteins	0,06	0,04	0,02	0,09	0,02	0,02
**109**	APOM	21,25	5,92	Lipoproteins	0,16	0,08	0,37	0,33	0,07	0,09
**110**	APOA	501,31	5,77	Lipoproteins	0,02	0,09	0,21	0,47	0,14	0,30
**111**	APOH	38,30	8,02	Lipoproteins	0,02	0,01	10,91	2,84	6,57	11,18
**112**	CD5L	38,09	5,14	Others	0,09	0,03	0,03	0,31	0,05	0,05
**113**	CD9	25,42	7,19	Others	0,00	0,00	0,00	0,01	0,00	0,01
**114**	HBA	15,26	9,09	Others	0,42	0,06	0,83	0,32	0,35	0,43
**115**	HBB	16,00	7,32	Others	0,49	0,14	1,42	0,22	0,23	0,31
**116**	HBE	16,20	9,11	Others	0,00	0,00	0,04	0,00	0,00	0,00
**117**	AMBP	39,00	6,15	Others	0,48	2,05	0,12	0,08	0,02	0,11
**118**	ALBU	69,37	6,21	Others	17,21	7,84	16,19	15,50	13,22	12,98
**119**	ACTS	42,05	5,05	Tissue leakage	0,00	0,00	0,00	0,01	0,01	0,02
**120**	ACTB	41,74	5,15	Tissue leakage	0,00	0,01	0,02	0,08	0,05	0,10
**121**	ACTBL	42,00	5,28	Tissue leakage	0,00	0,00	0,01	0,01	0,01	0,00
**122**	GELS	85,70	6,19	Tissue leakage	0,38	2,58	0,17	0,13	0,48	0,22
**123**	K1C10	59,51	4,88	Tissue leakage	0,02	0,00	2,04	1,52	1,91	0,94
**124**	K1C14	51,62	4,81	Tissue leakage	0,01	0,00	0,33	0,48	0,32	0,06
**125**	K1C15	49,17	4,42	Tissue leakage	0,00	0,00	0,00	0,00	0,00	0,00
**126**	K1C16	51,27	4,69	Tissue leakage	0,00	0,00	0,11	0,28	0,16	0,01
**127**	K1C17	48,11	4,67	Tissue leakage	0,00	0,00	0,02	0,08	0,03	0,00
**128**	K1C9	62,06	4,89	Tissue leakage	0,07	0,00	3,01	3,61	2,28	1,03
**129**	K2C1	66,04	8,33	Tissue leakage	0,20	0,01	4,65	5,01	3,45	1,86
**130**	K2C1B	61,36	5,78	Tissue leakage	0,00	0,00	0,00	0,00	0,00	0,00
**131**	K22E	65,43	8,17	Tissue leakage	0,01	0,00	0,97	0,54	0,78	0,36
**132**	K2C5	62,38	7,91	Tissue leakage	0,00	0,00	0,12	0,15	0,10	0,04
**133**	K2C6A	60,04	8,17	Tissue leakage	0,00	0,00	0,21	0,32	0,25	0,10
**134**	K2C6B	60,07	8,17	Tissue leakage	0,00	0,00	0,01	0,00	0,01	0,00
**135**	K2C73	58,92	7,53	Tissue leakage	0,00	0,00	0,00	0,01	0,01	0,00
**136**	K2C78	56,96	5,83	Tissue leakage	0,00	0,00	0,00	0,00	0,00	0,00
**137**	6PGL	27,55	5,94	Others	0,00	0,00	0,01	0,01	0,01	0,00
**138**	AFAM	69,07	5,70	Others	0,02	0,00	0,00	0,00	0,00	0,00
**139**	FETUA	39,34	5,53	Others	1,47	0,05	0,11	0,19	0,24	0,19
**140**	AMY2B	57,71	7,04	Others	0,00	0,00	0,00	0,00	0,00	0,00
**141**	ANGI	16,55	10,09	Others	0,00	0,00	0,09	0,00	0,08	0,14
**142**	ANGT	53,15	6,28	Others	0,04	0,09	0,02	0,01	0,02	0,01
**143**	ANXA2	38,60	7,42	Others	0,00	0,00	0,00	0,00	0,00	0,00
**144**	B3AT	101,79	4,84	Others	0,00	0,00	0,00	0,00	0,00	0,00
**145**	B2MG	13,71	6,50	Others	0,00	0,00	0,00	0,00	0,01	0,00
**146**	CNDP1	56,71	4,95	Others	0,00	0,05	0,00	0,00	0,00	0,00
**147**	BTD	61,13	6,22	Others	0,01	0,09	0,00	0,00	0,00	0,00
**148**	BPIB1	52,44	7,25	Others	0,00	0,00	0,00	0,01	0,00	0,00
**149**	BASP1	22,69	4,30	Others	0,00	0,00	0,00	0,00	0,00	0,00
**150**	C4BPA	67,03	7,31	Others	0,71	2,28	2,80	0,79	0,98	0,44
**151**	C4BPB	28,36	4,80	Others	0,05	0,29	0,19	0,01	0,07	0,02
**152**	CADH5	87,53	5,08	Others	0,00	0,01	0,00	0,00	0,00	0,00
**153**	CACO1	77,34	4,47	Others	0,00	0,00	0,00	0,01	0,01	0,01
**154**	CALL5	15,89	4,05	Others	0,00	0,00	0,05	0,12	0,04	0,05
**155**	CAH2	29,25	7,47	Others	0,00	0,00	0,03	0,00	0,00	0,00
**156**	CBPN	52,29	7,37	Others	0,00	0,00	0,00	0,00	0,05	0,02
**157**	CPN2	60,56	5,89	Others	0,00	0,00	0,00	0,00	0,00	0,00
**158**	CRAC1	71,42	4,72	Others	0,00	0,01	0,00	0,00	0,00	0,00
**159**	CILP1	132,56	8,48	Others	0,00	0,00	0,08	0,05	0,04	0,00
**160**	COMP	82,86	4,15	Others	0,00	0,06	0,00	0,00	0,00	0,00
**161**	CASPE	27,68	5,28	Others	0,00	0,00	0,00	0,00	0,00	0,00
**162**	CAMP	19,30	10,03	Others	0,00	0,00	0,00	0,00	0,01	0,02
**163**	CATD	44,55	6,50	Others	0,00	0,00	0,00	0,00	0,00	0,00
**164**	CCL18	9,85	8,90	Others	0,00	0,00	0,00	0,00	0,00	0,02
**165**	CD44	81,54	5,00	Others	0,00	0,00	0,00	0,00	0,00	0,00
**166**	CERU	122,20	5,50	Others	0,49	1,96	0,08	0,07	0,04	0,08
**167**	CETP	54,76	6,02	Others	0,00	0,00	0,00	0,00	0,00	0,00
**168**	CLUS	52,49	6,21	Others	0,48	2,68	0,62	0,75	0,97	1,10
**169**	COL11	28,67	5,05	Others	0,00	0,03	0,00	0,00	0,00	0,00
**170**	SPR1A	9,88	8,60	Others	0,00	0,00	0,01	0,03	0,02	0,00
**171**	CBG	45,14	5,94	Others	0,00	0,00	0,00	0,00	0,00	0,00
**172**	CRP	25,04	5,32	Others	0,00	0,00	0,01	0,00	0,00	0,01
**173**	CUL1	89,68	8,13	Others	0,00	0,00	0,00	0,00	0,01	0,00
**174**	CYTC	15,80	8,95	Others	0,00	0,00	0,00	0,00	0,02	0,00
**175**	DYHC1	532,40	6,34	Others	0,00	0,00	0,00	0,00	0,00	0,00
**176**	DCD	11,28	6,52	Others	0,01	0,00	0,61	0,20	0,44	0,47
**177**	DSC1	99,99	5,08	Others	0,00	0,00	0,00	0,01	0,00	0,00
**178**	DSG1	113,75	4,66	Others	0,00	0,00	0,00	0,00	0,00	0,00
**179**	DESP	331,77	6,78	Others	0,00	0,00	0,00	0,00	0,00	0,00
**180**	DUOX2	175,36	7,91	Others	0,00	0,01	0,00	0,00	0,00	0,00
**181**	BIP	72,33	4,80	Others	0,00	0,00	0,00	0,00	0,00	0,00
**182**	STOM	31,73	8,07	Others	0,00	0,00	0,00	0,00	0,00	0,01
**183**	ECM1	60,67	6,69	Others	0,01	0,00	0,00	0,00	0,00	0,00
**184**	FABP5	15,16	7,06	Others	0,00	0,00	0,00	0,07	0,01	0,02
**185**	URP2	75,95	6,99	Others	0,00	0,00	0,00	0,00	0,00	0,00
**186**	FBLN1	77,21	4,86	Others	0,01	0,00	0,00	0,00	0,00	0,00
**187**	FCN2	34,00	6,77	Others	0,00	0,00	0,00	0,02	0,00	0,00
**188**	FCN3	32,90	6,66	Others	0,00	0,00	0,00	0,00	0,00	0,00
**189**	FILA	435,16	9,60	Others	0,00	0,00	0,00	0,00	0,00	0,00
**190**	FILA2	248,07	8,35	Others	0,00	0,00	0,01	0,00	0,00	0,00
**191**	LG3BP	65,33	4,90	Others	0,00	0,00	0,00	0,00	0,00	0,00
**192**	GPX3	25,55	8,29	Others	0,01	0,01	0,02	0,05	0,11	0,05
**193**	G3P	36,05	8,73	Others	0,00	0,00	0,00	0,01	0,01	0,01
**194**	HEMO	51,68	7,02	Others	0,04	0,01	0,22	0,17	0,23	0,15
**195**	HEP2	57,07	6,90	Others	0,07	1,01	0,04	0,10	0,03	0,06
**196**	HGFA	70,68	7,23	Others	0,00	0,00	0,00	0,00	0,00	0,00
**197**	HGFL	80,32	7,70	Others	0,00	0,00	0,00	0,00	0,00	0,00
**198**	HRG	59,58	7,53	Others	0,02	0,08	0,07	0,03	0,96	0,09
**199**	1B59	40,58	6,25	Others	0,00	0,00	0,00	0,00	0,00	0,00
**200**	1C07	40,65	5,89	Others	0,00	0,00	0,00	0,00	0,00	0,00

**201**	HORN	282,39	10,25	Others	0,00	0,00	0,03	0,03	0,02	0,01
**202**	HABP2	62,67	6,53	Others	0,01	0,69	0,01	0,01	0,01	0,05
**203**	HYALP	57,85	7,00	Others	0,04	0,00	0,00	0,00	0,00	0,00
**204**	FCGBP	572,01	4,99	Others	0,00	0,00	0,00	0,00	0,00	0,00
**205**	IBP3	31,67	8,79	Others	0,00	0,00	0,00	0,00	0,02	0,01
**206**	IBP4	27,93	7,16	Others	0,00	0,00	0,00	0,00	0,00	0,00
**207**	IBP5	30,57	8,27	Others	0,00	0,00	0,00	0,00	0,00	0,00
**208**	ALS	66,03	6,78	Others	0,02	0,01	0,00	0,00	0,00	0,00
**209**	ITA2B	113,38	5,03	Others	0,00	0,00	0,00	0,00	0,00	0,01
**210**	ITB3	87,06	4,85	Others	0,00	0,00	0,00	0,00	0,00	0,01
**211**	ITLN1	34,96	5,89	Others	0,00	0,00	0,00	0,10	0,01	0,09
**212**	ITIH1	101,39	6,78	Others	0,22	2,02	0,11	0,09	0,03	0,11
**213**	ITIH2	106,46	6,85	Others	0,45	2,56	0,31	0,24	0,08	0,16
**214**	ITIH3	99,85	5,49	Others	0,03	0,64	0,00	0,00	0,00	0,01
**215**	ITIH4	103,36	6,97	Others	0,07	0,00	0,06	0,08	0,87	0,41
**216**	PLAK	81,74	6,05	Others	0,00	0,00	0,00	0,01	0,00	0,00
**217**	KAIN	48,54	7,85	Others	0,00	0,00	0,00	0,00	0,01	0,00
**218**	KTDAP	11,05	7,70	Others	0,00	0,00	0,00	0,00	0,00	0,01
**219**	KPRP	64,14	8,29	Others	0,00	0,00	0,01	0,02	0,01	0,00
**220**	KIF14	186,49	8,00	Others	0,00	0,00	0,00	0,00	0,00	0,00
**221**	LUM	38,43	6,61	Others	0,07	0,55	0,00	0,00	0,00	0,00
**222**	LYSC	16,54	9,36	Others	0,00	0,00	0,10	0,03	0,05	0,07
**223**	MASP1	79,25	5,18	Others	0,00	0,01	0,00	0,00	0,00	0,00
**224**	MBL2	26,14	5,14	Others	0,01	0,00	0,00	0,00	0,00	0,00
**225**	MA1A1	72,97	6,44	Others	0,00	0,01	0,00	0,00	0,00	0,00
**226**	CD14	40,08	6,16	Others	0,00	0,00	0,00	0,00	0,00	0,00
**227**	MUC19	805,24	4,65	Others	0,04	0,00	0,08	0,06	0,05	0,06
**228**	PGRP2	62,22	7,59	Others	0,01	0,00	0,00	0,02	0,01	0,01
**229**	DEF3	10,25	6,42	Others	0,00	0,00	0,02	0,06	0,09	0,03
**230**	NID1	136,38	4,93	Others	0,00	0,00	0,00	0,00	0,00	0,00
**231**	OBSL1	206,94	5,37	Others	0,00	0,00	0,00	0,00	0,00	0,00
**232**	VNN1	57,01	5,27	Others	0,00	0,01	0,00	0,00	0,00	0,00
**233**	PRDX2	21,89	7,04	Others	0,00	0,00	0,00	0,00	0,00	0,00
**234**	LCAT	49,58	6,05	Others	0,00	0,00	0,00	0,00	0,00	0,00
**235**	PHLD	92,34	6,33	Others	0,00	0,04	0,00	0,00	0,00	0,00
**236**	PLTP	54,74	7,02	Others	0,00	0,00	0,00	0,00	0,00	0,00
**237**	PEDF	46,31	6,33	Others	0,01	0,00	0,00	0,00	0,14	0,11
**238**	KLKB1	71,37	8,26	Others	0,00	0,00	0,00	0,00	0,16	0,12
**239**	IC1	55,15	6,52	Others	0,26	0,00	0,01	0,01	0,32	0,06
**240**	IPSP	45,67	9,77	Others	0,00	0,00	0,00	0,00	0,06	0,04
**241**	PLMN	90,57	7,25	Others	0,30	0,10	0,06	0,02	0,34	0,03
**242**	PLF4	10,85	9,13	Others	0,00	0,00	0,00	0,00	0,00	0,01
**243**	CD36	53,05	8,05	Others	0,00	0,00	0,00	0,00	0,00	0,00
**244**	GP1BA	71,54	6,24	Others	0,00	0,00	0,00	0,00	0,00	0,00
**245**	PCYOX	56,64	6,11	Others	0,00	0,00	0,00	0,00	0,00	0,00
**246**	PIP	16,57	8,19	Others	0,00	0,00	0,02	0,04	0,05	0,03
**247**	PROP	51,28	7,92	Others	0,00	0,00	0,06	0,13	0,02	0,08
**248**	S10A6	10,18	5,14	Others	0,00	0,00	0,00	0,00	0,00	0,00
**249**	S10A7	11,47	7,18	Others	0,00	0,00	0,00	0,05	0,03	0,01
**250**	S10A8	10,83	7,07	Others	0,00	0,00	0,04	0,17	0,10	0,03
**251**	S10A9	13,24	6,07	Others	0,00	0,00	0,12	0,22	0,20	0,05
**252**	ZPI	50,71	8,58	Others	0,00	0,07	0,00	0,00	0,01	0,01
**253**	PRG4	151,06	10,16	Others	0,00	0,00	0,00	0,00	0,05	0,11
**254**	GULP1	34,49	8,01	Others	0,00	0,00	0,01	0,00	0,00	0,00
**255**	RAP1A	20,99	5,40	Others	0,00	0,00	0,00	0,00	0,00	0,01
**256**	RARR2	18,62	9,47	Others	0,00	0,00	0,00	0,00	0,00	0,00
**257**	RET4	23,01	5,86	Others	0,23	0,02	0,03	0,03	0,05	0,02
**258**	PRPC	17,02	4,43	Others	0,00	0,00	0,01	0,00	0,03	0,01
**259**	SPP24	24,34	8,45	Others	0,00	0,04	0,00	0,00	0,00	0,00
**260**	SEPP1	43,17	7,92	Others	0,01	0,01	0,00	0,00	0,01	0,00
**261**	TRFE	77,06	7,13	Others	0,34	0,28	1,37	1,00	1,17	0,88
**262**	SAMP	25,39	6,52	Others	0,00	0,19	0,04	0,03	0,07	0,04
**263**	PON1	39,73	4,89	Others	0,14	0,97	0,26	0,24	0,34	0,44
**264**	PON3	39,61	5,11	Others	0,00	0,01	0,00	0,00	0,00	0,00
**265**	SPR2A	7,97	8,40	Others	0,00	0,00	0,00	0,06	0,00	0,00
**266**	SRCRL	165,74	6,06	Others	0,00	0,00	0,00	0,00	0,00	0,00
**267**	GTR1	54,08	8,92	Others	0,00	0,00	0,00	0,00	0,00	0,00
**268**	SBSN	60,54	7,02	Others	0,00	0,00	0,01	0,01	0,01	0,00
**269**	TETN	22,54	5,36	Others	0,03	0,01	0,00	0,00	0,25	0,11
**270**	TSP4	105,87	4,24	Others	0,00	0,00	0,00	0,00	0,00	0,00
**271**	TYB4	5,05	4,72	Others	0,00	0,00	0,00	0,01	0,00	0,00
**272**	FUCO	53,69	6,84	Others	0,00	0,00	0,00	0,00	0,00	0,00
**273**	TTHY	15,89	5,58	Others	0,44	0,31	0,40	0,18	0,43	0,39
**274**	TRIM6	56,40	7,57	Others	0,00	0,08	0,00	0,00	0,00	0,00
**275**	TRY3	32,53	7,54	Others	0,00	0,00	0,01	0,00	0,00	0,00
**276**	VTDB	52,92	5,21	Others	0,25	0,01	0,10	0,10	0,11	0,05
**277**	VTNC	54,31	5,54	Others	1,11	4,45	0,40	0,33	0,59	0,94
**278**	CAC1H	259,16	7,38	Others	0,00	0,00	0,00	0,00	0,00	0,00
**279**	VWF	309,26	5,16	Others	0,01	0,01	0,00	0,00	0,03	0,06
**280**	ZA2G	34,258	5,919	Others	0,00	0,00	0,01	0,01	0,01	0,01

Table 4. Functional classification of protein corona components. Relative protein abundance (RPA) of plasma proteins identified in the respective liposomes' coronas by quantitative nanoLC-MS/MS at the indicated human plasma concentrations. Plasma proteins were grouped according to biological processes of the blood system. Each value is the average of triplicates ± standard deviation within a single experiment.

Functional group	RPA (%)					
	DOTAP		DOPC		DOPG	
	HP=5%	HP=50%	HP=5%	HP=50%	HP=5%	HP=50%
**Acute Phase**	6.6±0.2	1.6±0.2	3.9±0.1	3.3±0.3	3.6±0.2	3.45±0.05
**Coagulation**	20±2	37±3	7.2±0.2	8±2	13±1	9±1
**Complement**	4±1	5±1	6.1±0.1	3.3±0.4	8.0±0.5	5.42±0.02
**Immunoglobulins**	14±2	6±2	17.9±0.7	16±5	13±5	9.6±0.4
**Lipoproteins**	29±11	15±10	25.8±0.3	35±11	28±12	46.3±0.8
**Tissue leakage**	0.7±2.9	3±2	11.7±0.3	12±4	10±4	4.7±0.1

Table 5. Relative protein abundance (RPA) of individual plasma proteins identified in the respective liposomes' coronas by quantitative nanoLC-MS/MS at the indicated human plasma concentrations. Each value is the average of triplicates ± standard deviation within a single experiment.

Protein	RPA (%)					
	DOTAP		DOPC		DOPG	
	HP=5%	HP=50%	HP=5%	HP=50%	HP=5%	HP=50%
AMBP	0.5±0.1	2.05±0.01	0.12±0.01	0.08±0.01	0.017±0.06	0.11±0.08
ALBU	17.21±0.01	7.84±0.02	16.19±0.02	15.50±0.1	13.22±0.01	12.98±0.01
FETUA	1.5±0.1	0.045±0.007	0.11±0.01	0.19±0.01	0.24±0.03	0.19±0.01
C4BPA	0.71±0.06	2.28±0.03	2.8±0.03	0.79±0.06	0.98±0.04	0.442±0.002
C4BPB	0.051±0.002	0.29±0.02	0.186±0.002	0.006±0.001	0.070±0.002	0.021±0.003
CERU	0.49±0.02	1.96±0.07	0.081±0.002	0.074±0.002	0.043±0.003	0.085±0.004
CLUS	0.48±0.02	2.7±0.1	0.62±0.01	0.75±0.02	0.97±0.03	1.1±0.4
HEP2	0.071±0.003	1.01±0.04	0.041±0.002	0.101±0.001	0.031±0.001	0.064±0.002
HRG	0.022±0.002	0.081±0.006	0.0712±0.003	0.032±0.002	0.96±0.02	0.090±0.004
ITIH1	0.22±0.03	2.0±0.5	0.11±0.03	0.09±0.01	0.0265±0.006	0.112±0.004
TRFE	0.343±0.002	0.281±0.005	1.37±0.03	1.00±0.06	1.17±0.07	0.88±0.02
PON1	0.141±0.003	0.967±0.004	0.262±0.001	0.237±0.006	0.338±0.003	0.441±0.005
VTNC	1.11±0.05	4.45±0.09	0.402±0.001	0.332±0.003	0.589±0.006	0.938±0.005
