# Supplementary material for: Interplay of protein corona and immune cells controls blood residency of liposomes
Source: Nat Commun. 2019 Aug 15;10:3686. doi: 10.1038/s41467-019-11642-7 (PMC6695391; doi:10.1038/s41467-019-11642-7)
Supplement: Supplementary file 3 — Reporting Summary [file 41467_2019_11642_MOESM3_ESM.pdf]

nature research

Corresponding author(s): NCOMMS-19-01850B

Last updated by author(s): Jul 14, 2019

Reporting Summary

Nature Research wishes to improve the reproducibility of the work that we publish. This form provides structure for consistency and transparency in reporting. For further information on Nature Research policies, see Authors & Referees and the Editorial Policy Checklist.

Statistics

For all statistical analyses, confirm that the following items are present in the figure legend, table legend, main text, or Methods section.

n/a	Confirmed	
☐	☒	The exact sample size (n) for each experimental group/condition, given as a discrete number and unit of measurement
☐	☒	A statement on whether measurements were taken from distinct samples or whether the same sample was measured repeatedly
☒	☐	The statistical test(s) used AND whether they are one- or two-sided<br>*Only common tests should be described solely by name; describe more complex techniques in the Methods section.*
☒	☐	A description of all covariates tested
☒	☐	A description of any assumptions or corrections, such as tests of normality and adjustment for multiple comparisons
☐	☒	A full description of the statistical parameters including central tendency (e.g. means) or other basic estimates (e.g. regression coefficient) AND variation (e.g. standard deviation) or associated estimates of uncertainty (e.g. confidence intervals)
☒	☐	For null hypothesis testing, the test statistic (e.g. F, t, r) with confidence intervals, effect sizes, degrees of freedom and P value noted<br>*Give P values as exact values whenever suitable.*
☒	☐	For Bayesian analysis, information on the choice of priors and Markov chain Monte Carlo settings
☒	☐	For hierarchical and complex designs, identification of the appropriate level for tests and full reporting of outcomes
☒	☐	Estimates of effect sizes (e.g. Cohen's d, Pearson's r), indicating how they were calculated

Our web collection on statistics for biologists contains articles on many of the points above.

Software and code

Policy information about availability of computer code

Data collection	ZetaSizer Ultra; FlowJo v10 LLC data analysis software, Ashland, OR, USA;
Data analysis	ZetaSizer Ultra; FlowJo v10 LLC data analysis software, Ashland, OR, USA; Xcalibur software (version 2.2 SP1.48, Thermo Fisher Scientific)

For manuscripts utilizing custom algorithms or software that are central to the research but not yet described in published literature, software must be made available to editors/reviewers. We strongly encourage code deposition in a community repository (e.g. GitHub). See the Nature Research guidelines for submitting code & software for further information.

Data

Policy information about availability of data

All manuscripts must include a data availability statement. This statement should provide the following information, where applicable:

- Accession codes, unique identifiers, or web links for publicly available datasets
- A list of figures that have associated raw data
- A description of any restrictions on data availability

The datasets generated during and/or analysed during the current study are available from the corresponding author on reasonable request

Field-specific reporting

Please select the one below that is the best fit for your research. If you are not sure, read the appropriate sections before making your selection.

☒ Life sciences ☐ Behavioural & social sciences ☐ Ecological, evolutionary & environmental sciences

For a reference copy of the document with all sections, see nature.com/documents/nr-reporting-summary-flat.pdf

Life sciences study design

All studies must disclose on these points even when the disclosure is negative.

Sample size	no sample-size calculation was performed
Data exclusions	no data were excluded from the analysis
Replication	Attempts at replication were successful
Randomization	n/a
Blinding	n/a

Reporting for specific materials, systems and methods

We require information from authors about some types of materials, experimental systems and methods used in many studies. Here, indicate whether each material, system or method listed is relevant to your study. If you are not sure if a list item applies to your research, read the appropriate section before selecting a response.

Materials & experimental systems

n/a	Involved in the study	
☒	☐	Antibodies
☐	☒	Eukaryotic cell lines
☒	☐	Palaeontology
☒	☐	Animals and other organisms
☐	☒	Human research participants
☒	☐	Clinical data

Methods

n/a	Involved in the study	
☒	☐	ChIP-seq
☐	☒	Flow cytometry
☒	☐	MRI-based neuroimaging

Eukaryotic cell lines

Policy information about cell lines

Cell line source(s)	THP1 cells were purchased from ATCC. Peripheral blood mononuclear cells (PBMCs) were freshly isolated from peripheral blood samples of anonymized volunteer healthy donors of Transfusion Center of Sapienza University of Rome
Authentication	we used newly purchased cells
Mycoplasma contamination	All lines tested were negative for mycoplasma contamination
Commonly misidentified lines (See ICLAC register)	n/a

Human research participants

Policy information about studies involving human research participants

Population characteristics	20-40 years old, male, healthy donors
Recruitment	Peripheral blood mononuclear cells (PBMCs) were freshly isolated from peripheral blood samples of anonymized volunteer healthy donors of Transfusion Center of Sapienza University of Rome
Ethics oversight	Written informed consent was obtained from blood donors, and both the informed consent form and procedure were approved by the Ethics Committee of Sapienza University of Rome. The study was conducted in accordance with the Declaration of Helsinki

Note that full information on the approval of the study protocol must also be provided in the manuscript.

Flow Cytometry

Plots

Confirm that:

☒ The axis labels state the marker and fluorochrome used (e.g. CD4-FITC).

☒ The axis scales are clearly visible. Include numbers along axes only for bottom left plot of group (a 'group' is an analysis of identical markers).

☒ All plots are contour plots with outliers or pseudocolor plots.

☒ A numerical value for number of cells or percentage (with statistics) is provided.

Methodology

Sample preparation	Peripheral blood mononuclear cells (PBMCs) were freshly isolated from peripheral blood samples of anonymized volunteer healthy donors of Transfusion Center of Sapienza University of Rome
Instrument	BD LSFORTESSA (BD Biosciences, San Jose, CA, USA)
Software	FlowJo LLC data analysis software v10, Ashland, OR, USA
Cell population abundance	sorting was not performed
Gating strategy	After the treatment, cells were washed with PBS and then labeled with the antibodies anti-CD3/BV510, CD56/BV421, an-ti-CD4/ APC, anti-CD14/PerCP, anti- anti-CD45/allophycocyanin-H7), and anti-CD19/PE-Cy7 (BD Bioscience)

☒ Tick this box to confirm that a figure exemplifying the gating strategy is provided in the Supplementary Information.
